# Editorial: Neurosonology in stroke medicine and neurocritical care

**DOI:** 10.3389/fneur.2024.1380588

**Published:** 2024-02-14

**Authors:** Stefano Caproni, Piergiorgio Lochner, Michele Romoli

**Affiliations:** ^1^Neurology and Stroke Unit, Neuroscience Department, “S. Maria” University Hospital, Terni, Italy; ^2^Department of Neurology, Saarland University Medical Center, Homburg, Germany; ^3^Neurology and Stroke Unit, Department of Neuroscience, Bufalini Hospital, Cesena, Italy

**Keywords:** Neurosonology, transcranial color code sonography (TCCS), stroke, neurocritical care, predictor

In the dynamic landscape of stroke medicine and neurocritical care, Neurosonology increasingly plays a pivotal role in unraveling the complexities of cerebrovascular diseases and providing real-time insights that influence patient outcomes. The transcranial color sonology technique (TCCS) was introduced clinically in the 1990 by pioneering studies (1, 2). Although limited sometimes qualitatively by the cranial bone, TCCS is able to provide both a high resolution of echogenic deep brain structures in the focal zone of transducer and to ensure adequate imagine of the circle of Willis in at least 70% of subjects. In the remaining cases, the use of contrast-enhanced ultrasound can be applied. Furthermore, the latest technological innovations, such as the superb microvascular imaging, promise to overcome the current technical limitations, making it possible to deepen the neurosonological study down to the level of the intracranial microcirculation and the neovascularisation of atheromatous plaques (3). TCCS offers a unique window into cerebral hemodynamics, going beyond anatomical delineation, delving into the functional aspects that underpin critical the management of patients in stroke units and intensive care units. At the heart of Neurosonology's significance lies its patient-centric approach. By offering non-invasive, bedside assessments, Neurosonology minimizes the burden on patients while maximizing the depth of information for healthcare providers. This accessibility aligns with the ethos of patient-centered care, ensuring that diagnostic and therapeutic decisions are rooted in the individual needs and nuances of each patient (4).

In this topic we consider the application of Neurosonology in the detection of stroke prevention, acute stroke diagnosis and early management and, moreover, the usefulness of Neurosonology in intensive care medicine, particularly vasospasm detecting and monitoring, critical illness assessment and cerebral death confirmation.

In stroke medicine, time is always brain, also outside the hyperacute phase. The ability of Neurosonology to swiftly and non-invasively assess blood flow velocity, detect stenosis, and monitor collateral circulation provides a valuable dimension to the diagnostic timeline. Early identification of high-risk features through Neurosonology expedites intervention in strokes of various etiologies (5). Furthermore, Neurolosonolgy follow-up enhances the precision of therapeutic strategies, adding predictive information (6).

The precise definition of the degree of stenosis of the internal carotid artery and which diagnostic tool and criteria can be exploited are still under discussion in the literature. Pakizer et al. found that that not only the assessment of peak systolic velocity (PSV) alone, but also other parameter such as PSV ratio in stenosis and distal to stenosis (PSVICA/ICA ratio), end-diastolic velocity (EDV), and B-mode may have shown comparable correlations with computed tomography angiography (CTA) in stenosis assessment. The authors identified the severe degree of calcifications and the irregularity of the surface of the as the limitation of stenosis definition. In their meta-analysis Szegedi et al. showed that standard duplex ultrasound criteria could overestimate the degree of restenosis after carotid stenting (CAS) and after carotid endarterectomy (CEA). Therefore, the authors concluded that PSV threshold values after CAS were to be higher than after carotid endarterectomy CEA for estimating ≥50 and ≥70% restenosis. Moreover, the authors suggested best medical therapy in case of ICA restenosis after invasive procedures, because of lack of complications and low rate of ischemic strokes. The authors indicated that prospective studies should be designed to identify which individuals are at high risk of stroke in case of restenosis.

In the post-acute phase, Neurosonology can contribute to better understanding the mechanisms underlying the efficacy of reperfusion treatment. In particular, the detection of ipsilateral or contralateral carotid stenosis can explain a worse outcome due to, respectively, hypoperfusion/continuous microembolization and reduced effectiveness of collateral circles (Viticchi et al.).

Moving to neurocritical care medicine, TCCS can represent an indispensable instrument for evaluating the hemodynamic consequences of vasospasm, assessing collateral flow, and forecasting the potential for recurrence damage, in several critical illnesses (subarachnoid hemorrhage, traumatic brain injury, post anoxic encephalopathy). This real-time feedback loop enables healthcare providers to tailor interventions and management strategies with unprecedented precision (7).

In the current Research Topic, two studies address the prediction and prognostication of patients in the neurointensive care unit through the implementation of Neurosonology. In a sub-study of the prospective Norwegian Cardiorespiratory Arrest Study from Reichenbach et al., TCD was implemented at day 1 through day 7 at regular intervals to define the potential value in outcome prediction. Among 139 patients evaluated after resuscitation for out-of-hospital cardiac arrest, PSV in the middle cerebral artery was low during temperature control (Day 1) and increased after rewarming (Day 3). A subsequent rise in PSV identified patients with poor outcome, while people with good functional outcome, defined as cerebral performance category CPC 1–2, tended to have stable PSV values over follow-up (Reichenbach et al.). Serial TCD during the first week can therefore be implemented to refine prognostication after cardiac arrest.

Sepsis has high prevalence among intensive care unit (ICU) patients, with sepsis-associated encephalopathy lacking prediction instruments and prognostication. In this Research Topic, Mei et al. from the Shanghai University (China) tested the accuracy of cerebral circulation time (CCT) with contrast-enhanced ultrasound CEUS in the prediction of sepsis-associated encephalopathy. Among 62 patients in the ICU diagnosed with sepsis within 24 h, CCT had an 85% accuracy in predicting the development of encephalopathy, over-performing pulsatility index and S100B biomarker. A normogram based on all three variables is provided, which can be applied for external validation (Mei et al.).

Furthermore, another potential usefulness of Neurosonology in neurocritical care has been shown by Gelormini et al., highlighting that serial TCCS can detect hyperemia, that is associated with the risk of intracranial hypertension in patients with traumatic brain injury. In light of these results, TCCS promises to play an increasingly important role in the complicated management of patients in ICU.

In conclusion, the symbiotic relationship between Neurosonology, stroke medicine, and neurocritical care exemplifies the synergy that emerges when medical expertise and technological prowess converge. As we navigate the future, the harmonious integration of Neurosonology into routine practice promises to elevate the standard of care for patients grappling with cerebrovascular challenges. Through collaborative efforts, research initiatives, and a commitment to patient-centricity, Neurosonology stands poised as an instrumental force in the ongoing narrative of advancements in neurological healthcare.

## Author contributions

SC: Writing – original draft, Writing – review & editing. PL: Writing – original draft, Writing – review & editing. MR: Writing – original draft, Writing – review & editing.
